# A Case of Mania Cured

**Published:** 1803-07-01

**Authors:** 

**Affiliations:** W. Hampton, Staffordshire


*A* Case *of* Mania *cured; communicated by* Mr. Hickin, *of W. Hampton, Staffordshire.*

Mary Allen, aged about twenty, a few days before the 23d of last February, was attacked with Madness. On the 23d I was requested to see her, and found her as follows: She was raving, and so inconsistently that I could not perceive what was predominant in her mind; her pulse was upwards of 70; menses regular; she would sing, bow, laugh, and cry. Inquiry was made, to endeavour to find the remote cause, but no satisfactory decision could be made. The regimen ordered was bread and water for about the first three weeks, and then, by degrees, a more nourishing diet. The treatment was the antiphlogistic, as,

On February 23, V. sectio ℔j. ℞. Antim. tart. gr. vj. kali ℈j. aquæ puræ, ℥j. M. ft. haust vesp. sumend. This did not operate.

On the 24th, the following, ℞. Antim. tart. gr. vj. in ch. ppt. vj. j. 4tis horis sumend. which occasioned no sickness so as to operate. ℞. Opii. crud. saponis ā gr. j. ft. pil. h. s. sumend.

On the 25th, ℞. Pulv. sal. nitri. ʒij. antim. tart. gr. xij. M. in. ch. vj. j. 4tis horis sumend. ℞. Pil. ut heri h. s. s.

On the 26th, The powders as yesterday. She had on this day a purging, with lowness of spirits.

On the 27th, ℞. Cort. cascarill. ℥ß. coque in aq. ℔ß. pro min. 10, cola, et capiat. coch. larg. ij. 4tis horis. This evening she begun to mend very much, and every night she had an opiate as above, which from the first time it was employed had a good effect.

On March 1, she was attacked with symptoms of Influenza, for which she took the following: ℞. Pulv. ipecac. ℈j. antim. tart. gr. vj. M. ft. pulv.; this operated very well. ℞. Pil. opii h. s. sumend.

March 2, Complains of a violent pain in the head with throbbing. ℞. Emp. cantharid. capit. applicand; this gave her a good deal of trouble.

A few days after she complained of great lowness, for which two compound galbanum pills were given every four hours. Continues recovering; is taken out a walking, and recovering without any thing farther, save an opiate every night for a few weeks; and is on this day, May 14, 1803, as well as ever she was in her life.

